# Dynamin-like Protein 1 (DNML1) as a Molecular Target for Antibody-Based Immunotherapy to Treat Glaucoma

**DOI:** 10.3390/ijms232113618

**Published:** 2022-11-07

**Authors:** Henrik Tonner, Selina Hunn, Nadine Auler, Carsten Schmelter, Norbert Pfeiffer, Franz H. Grus

**Affiliations:** Department of Experimental Ophthalmology, University Medical Center, Johannes Gutenberg University, Langenbeckstr. 1, 55131 Mainz, Germany

**Keywords:** glaucoma, antibodies, neuroprotection, neurodegeneration, discovery proteomics, dynamin-related protein 1, DRP1, DNM1L, animal model

## Abstract

Slow and progressive loss of retinal ganglion cells (RGCs) is the main characteristic of glaucoma, the second leading cause of blindness worldwide. Previous studies have shown that impaired mitochondrial dynamics could facilitate retinal neurodegeneration. Mitochondrial dynamics are regulated directly (fission) or more indirectly (fusion) by dynamin-like protein 1 (DNML1). Therefore, DNM1L might be a promising target for an antibody-based approach to treat glaucoma. The consequences of targeting endogenous DNM1L by antibodies in a glaucoma animal model have not been investigated yet. Here, we show that the intravitreal application of an anti-DNM1L antibody showed protective effects regarding the survival of RGCs and their axons in the retinal nerve fiber layer (RNFL). Antibody treatment also improved retinal functionality, as observed by electroretinography (Ganzfeld ERG). Western blot analysis revealed altered DNM1L phosphorylation and altered expression of proteins related to apoptosis suggesting a decreased apoptosis rate. Mass spectrometry analysis revealed 28 up-regulated and 21 down-regulated proteins (*p* < 0.05) in both experimental groups. Protein pathway analysis showed that many proteins interacted directly with the target protein DNM1L and could be classified into three main protein clusters: Vesicle traffic-associated (NSF, SNCA, ARF1), mitochondrion-associated (HSP9A, SLC25A5/ANT2, GLUD1) and cytoskeleton-associated (MAP1A) signaling pathway. Our results demonstrate that DNM1L is a promising target for an antibody-based approach to glaucoma therapy.

## 1. Introduction

Glaucoma comprises a group of ocular diseases in which primary open-angle glaucoma (POAG) is the most common form [1]. The disease accompanies various pathophysiological processes that contribute to the apoptosis of retinal ganglion cells (RGCs), leading to vision loss and complete blindness in the patients concerned. Glaucoma is, after age-related macular degeneration, the second leading cause of blindness worldwide, with increasing prevalence [2,3]. A major risk factor and the only therapeutic target in clinical practice is elevated intraocular pressure (IOP). However, since only about 70% of glaucoma patients possess an elevated IOP, disease progression still occurs in patients despite successful IOP-lowering therapy [4,5]. Therefore, it is likely that molecular mechanisms underlying RGC loss in addition to high IOP, such as glutamate excitotoxicity, abnormal protein accumulation, vascular dysfunction, and oxidative stress [6,7,8,9,10]. Increasing IOP can also initiate processes such as oxidative stress [11]. In recent years, oxidative stress has focused more on therapeutic approaches to treat glaucoma [12,13]. One consequence, especially with prolonged oxidative stress, is the alteration of mitochondrial dynamics which often occurs in other neurodegenerative diseases, such as Alzheimer’s (AD) or Parkinson’s disease (PD) [14,15].

Dynamin-related protein 1 (DRP1) or dynamin-1-like protein (DNM1L) is a small GTPase that regulates mitochondrial dynamics, i.e., fission processes but also indirect fusion processes, to affect the size, shape, and distribution of neurons [16]. Since neuronal cells, in particular, have a high energy demand, fission and fusion must be very tightly regulated. An imbalance of these processes can lead to abnormal structures, leading to mitochondria dysfunctions, such as disturbed ATP production [16]. Consequently, reactive oxygen species (ROS) may increase, leading to the apoptosis of neurons. Dysregulation of DNM1L-dependent mitochondrial fission is associated with neurodegenerative processes in diseases such as AD, Huntington’s, PD, or glaucoma [17,18,19]. DNM1L activity is modulated by various post-translational modifications (PTMS), such as phosphorylation, ubiquitination, sumoylation, and S-nitrosylation [17,20]. The pathological imbalance between the DNM1L phosphorylation sites DNM1Lp635 and DNM1Lp656 might contribute to the pathogenesis of various neurodegenerative diseases with a special focus on glaucoma [21].

The present study aimed to investigate the structural and physiological effects of anti-DNM1L Ab injection in a model of chronic IOP elevation. For this purpose, the IOP, the retinal nerve fiber layer thickness (RNFLT), and the retinal functionality were investigated in vivo. In addition, RGC density was determined post-mortem. Changes in DNM1L expression and phosphorylation sites were investigated by western blots, like the expression of the proteins XIAP and BAD, which are involved in mitochondrial apoptosis. In addition, the retinal proteome was analyzed by mass spectrometry to capture the effects of anti-DNM1L Ab treatment in a larger molecular context, which may allow conclusions to be drawn about potential antibody-induced neuroprotective mechanisms. We suggest that intravitreal anti-DNM1L injection may alter DNM1L activity, possibly by changing the ratio of phosphorylation sites, and may cause less apoptosis via the mitochondrial signaling pathway.

## 2. Results

### 2.1. Episcleral Vein Occlusion Chronically Elevates the Intraocular Pressure (IOP)

**Figure 1:**
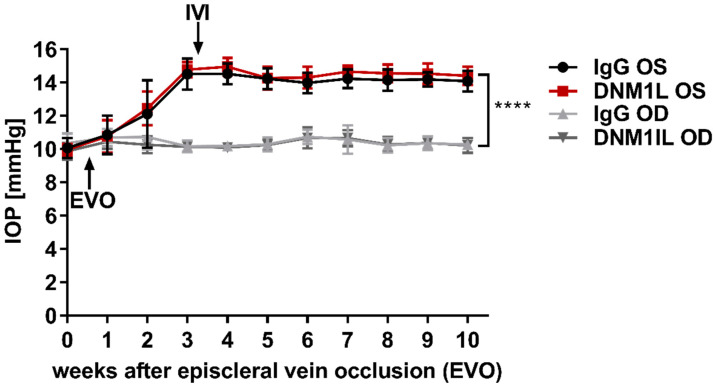
Longitudinal IOP monitoring. Rebound tonometry was used to examine IOP once a week. IOP was stably elevated three weeks after episcleral vein occlusion (EVO) at the OS. Stable IOP elevation was followed by intravitreal injection (IVI) of either IgG control antibody (IgG OS, Black) or anti-DNM1L antibody (DNM1L OS, Red). The contralateral OD, IgG OD (light gray), and DNM1L OD (dark gray), received no interventions and served as internal controls. IOP remained stably elevated until the end of the study. The increase in w10 was statistically significant. **** *p* < 0.0001, student’s *t*-test. Numbers of animals used: *n* = 7 (IgG), *n* = 8 (DNM1L).

Episcleral vein occlusion (EVO) was performed on the OS to increase the intraocular pressure (IOP) to induce glaucoma-like conditions; OD served as an internal control. EVO increased the IOP of the OS to 14.5 ± 0.9 mmHg in w3 in one study group, which subsequently received the IgG control Ab injected intravitreally into the OS (Figure 1). In another study group, the IOP of the OS increased to 14.8 ± 0.5 mmHg in w3, which received an anti-DNM1L Ab intravitreally injected into the OS immediately after the examination at w3 (DNM1L OS). By w10, IOP remained significantly elevated in the IgG OS group at 14.1 ± 0.6 mmHg and the DNM1L OS group at 14.4 ± 0.6 mmHg compared with the respective OD groups. The IOP of the OD groups at w0 was 10.4 ± 0.6 mmHg (IgG OD) and 9.9 ± 0.5 mmHg (DNM1L OD), respectively. Similarly, in w10, the IOP was 10.3 ± 0.1 mmHg in the IgG OD and 10.2 ± 0.4 mmHg in the DNM1L OD group. EVO chronically and significantly increased the IOP of the operated OS in both study groups.

### 2.2. Attenuated Retinal Ganglion Cell (RGC) Loss by Anti-DNM1L Ab Injection

**Figure 2:**
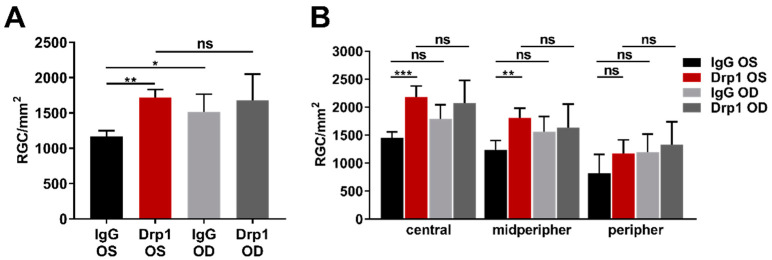
Quantitative analysis of Brn3^+^ RGCs in retinal flatmounts. Retinal ganglion cells were stained using immunostaining of Brn3a. An anti-Brn3a antibody (C20, Santa Cruz, Dallas, TX, USA) was used as the primary antibody and an anti-goat AF568 antibody (Life Technologies, Carlsbad, CA, USA) as the secondary antibody. Single images were taken with a fluorescence microscope (Eclipse TS 100 microscope, Nikon, Yurakucho, Tokyo, Japan), DS-Fi1-U2 digital microscope camera (Nikon, Tokyo, Japan). Objective: ELWD 20×/0.45 S Plan Flour Ph1 ADM Air objective (Nikon, Tokyo, Japan). (**A**) Nine images were taken from each flatmount. The RGC number was determined for each image. The average RGC number of the whole flatmount was calculated over all nine images. (**B**) Three of the total nine images were taken for each of the areas central, mid-peripheral, peripheral. For each area, the average RGC density was calculated over the three recordings of one area. * *p* < 0.05, ** *p* < 0.01, *** *p* < 0.001, ns = not significant, One-way ANOVA, Tukey’s HSD test (post hoc). Numbers of animals used: *n* = 7 (IgG), *n* = 8 DNM1L.

**Figure A1:**
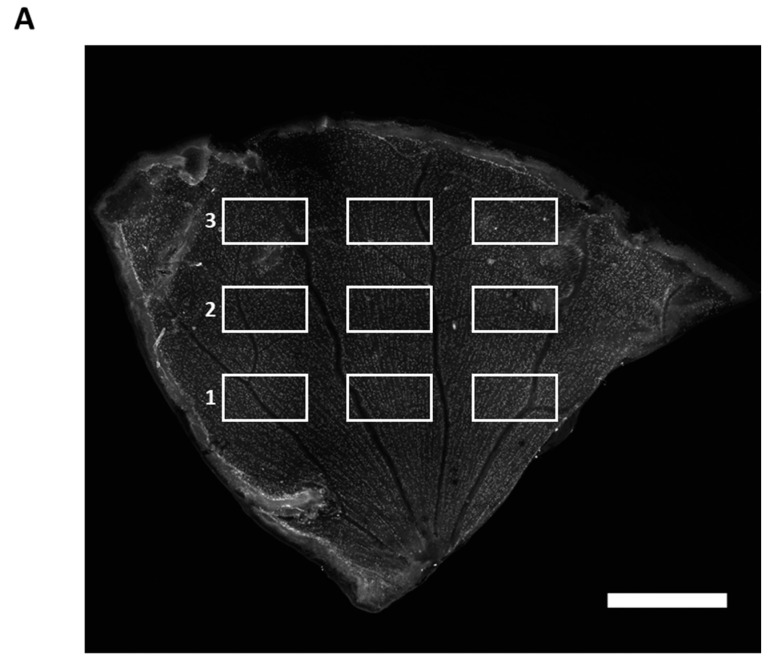

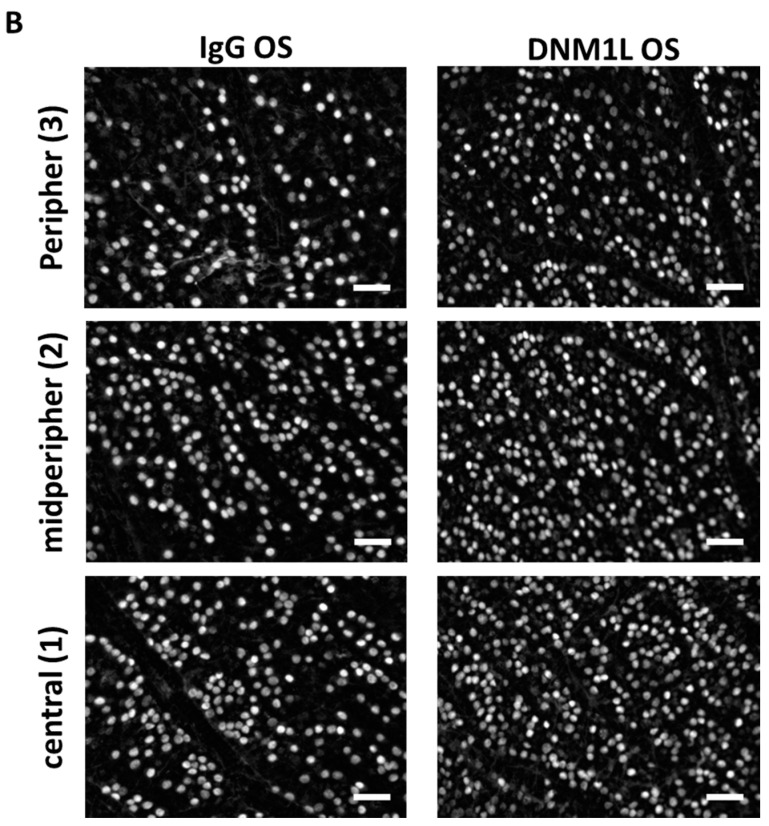
Immunofluorescence staining of RGCs with Brn3a. (**A**) Example of a retinal flatmount with RGCs stained by indirect immunofluorescence of Brn3a. The flatmount is divided into three sections: central (1), mid-periphery (2), and periphery (3). Scale bar: 1 mm. Three images are recorded within each region. Calculating the RGC count was performed by summing up the individual count of each image and then dividing by the number of images included. For regional analysis of the RGC count, the RGC count of three images of each region is summed up and divided by the number of images used for calculation. (**B**) Representative Brn3a stained images of each region. IgG OS group (left panel), DNM1L OS group (right panel). Scale bar: 50 µm.

To examine the retinal damage caused by IOP elevation more closely, RGCs were stained and quantified by immunostaining with the established marker brain-specific homeobox/POU domain protein 3A (Brn3a, Figure A1). The average RGC density of the whole flatmount was 1169 ± 78 RGC/mm^2^ in the IgG OS group (Figure 2A). In the DNM1L group, RGC density was significantly higher, with 1723 ± 110 RGC/mm^2^, compared to the IgG OS group (*p* < 0.01). The RGC densities were 1519 ± 250 RGC/mm^2^ (IgG OD) and 1682 ± 370 RGC/mm^2^ (DNM1L OD). The RGC density of the IgG OD group was significantly higher compared with the IgG OS group (*p* < 0.05), and the comparison of the DNM1L OS and OD showed no significant difference (ns).

In this study, the RGC density of the whole flatmount and the regional areas central, mid-peripheral and peripheral were quantified (Figure 2B). The DNM1L OS group had a significantly higher RGC density in the central area with 2180 ± 199 RGC/mm^2^ compared to 1448 ± 112 RGC/mm^2^ in the IgG group (*p* < 0.001). The IgG OD group had an RGC density of 1789 ± 253 RGC/mm^2^, and the DNM1L group had 2074 ± 406 RGC/mm^2^, which was not significantly altered compared with the RGC densities in the corresponding OS groups.

Like in the central region, the DNM1L OS group had significantly higher RGC density in the mid-peripheral with 1812 ± 170 RGC/mm^2^ compared with 1238 ± 169 RGC/mm^2^ in the IgG OS group (*p* < 0.01). The DNM1L OD group had an RGC density of 1639 ± 415 RGC/mm^2^, and the IgG OS group had 1564 ± 270 RGC/mm^2^, which were not significantly changed compared with the corresponding OS groups.

In the peripheral area, the DNM1L OS group had an RGC density of 1176 ± 240 RGC/mm^2^, and the DNM1L OD group had an RGC density of 1333 ± 406 RGC/mm^2^. The IgG OS group had an average RGC density of 822 ± 334 RGC/mm^2^, and the IgG OD group had an RGC density of 1202 ± 317 RGC/mm^2^. In the peripheral area, RGC densities were not significantly changed.

Overall, intravitreal injection of anti-DNM1L Ab alleviated the loss of RGCs in individual regions

### 2.3. The Intravitreal Injection of an Anti-DNM1L Ab Reduced the RNFL Thickness Loss

**Figure 3:**
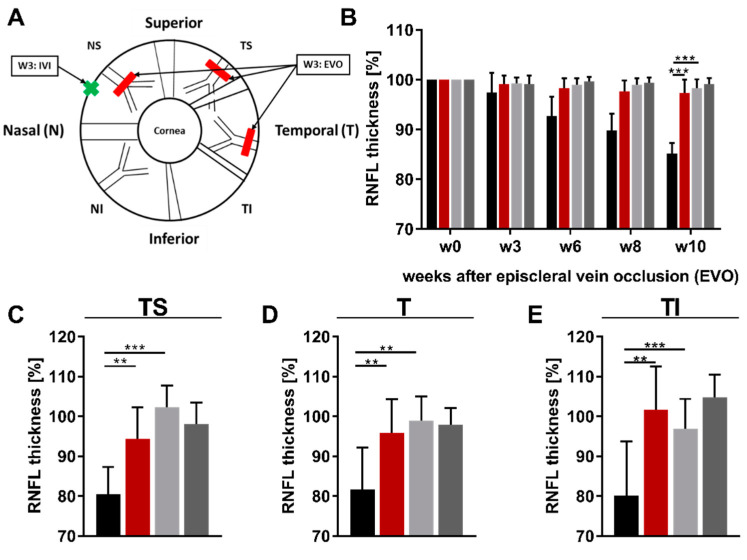

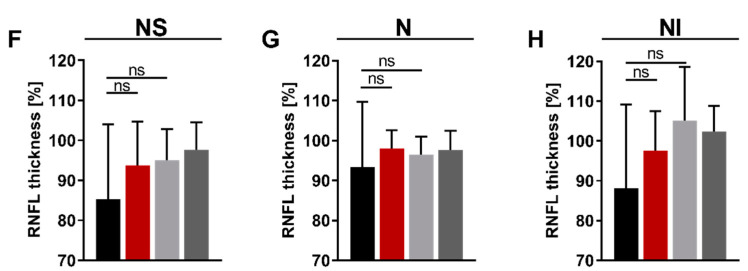
Retinal nerve fiber layer (RNFL) thickness analysis. Optical coherence tomography (OCT) was used to scan the retinal cross-sections in vivo. OCT allowed the acquisition of different retinal layers. For the analysis of the acquired circular B-scans with 12° diameter, a semi-automatic algorithm was used to define the RNFL and calculate its thickness (RNFLT). The Heidelberg Eye Explorer software divided the scan into the main sectors superior (S), temporal (T), inferior (I), and nasal (N). Furthermore, the sectors temporal superior (TS), temporal inferior (TI), nasal inferior (NI), and nasal superior (NS) were included (**A**). In addition to the regional classification, the schematic illustration also showed the sides of the interventions, episcleral vein occlusion (EVO) and intravitreal injection (IVI), which were performed on the eye during the study (Scheme modified from [22]. The mean RNFLT of the whole B-scan was used for follow-up quantification (**B**). Further quantification of the RNFLT at week 10 was performed for the individual sectors (**C**–**H**). Legend: black (IgG OS), red (DNM1L), light grey (IgG OD), dark grey (IgG OD), ** *p* < 0.01, *** *p* < 0.001, ns = not significant; One-way ANOVA, Tukey’s HSD test (post hoc). Numbers of animals used: *n* = 7 (IgG), *n* = 8 DNM1L.

**Figure A2:**
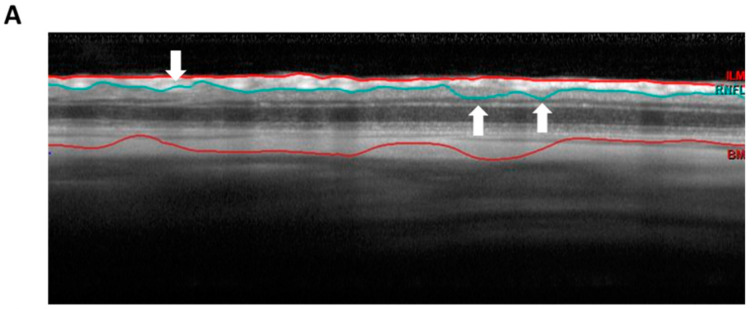

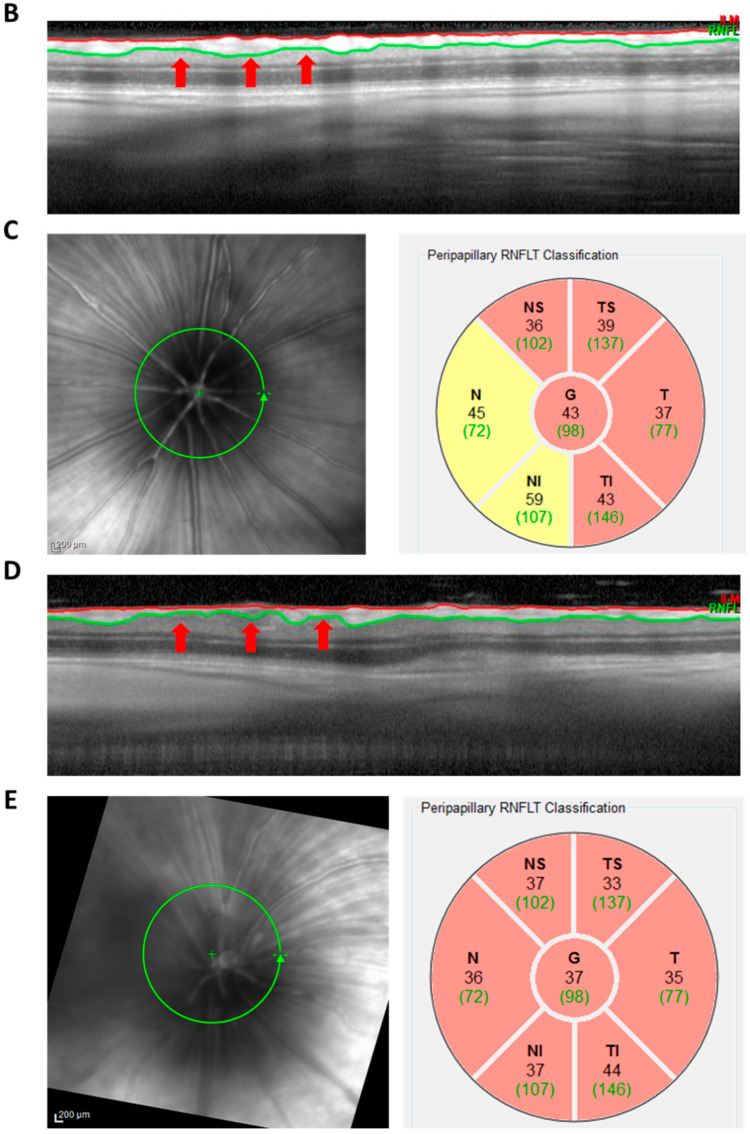
Analysis of OCT images. The OCT is acquired in the form of circular B-scans with 12° diameter. The B-scan consists of 1536 A-scans (cross-sections) acquired along the circle. The linear cross-section shown is the consecutive sequence of these A-scans. The RNFLT is identified in the first step by an algorithm which was developed for human eyes and therefore requires manual adjustment (white arrows, **A**). This results in a defined region marked by the inner limiting membrane (red) and the lower border of the RNFL (green) (**B**). The B-scan shown is the baseline examination of the OS of the IgG group. (**C**) RNFL quantification of the region shown in (**B**). The circular B-scan around the peripapillary region (**C**, left) is divided by the Heidelberg Eye Explorer software into six segments, for each of which the RNFL thickness is calculated (**C**, right). In addition, the average total RNFL thickness is calculated (**C**, right, G). (**D**) B-scan of week 10 examination of the same animal shown in (**B**). The red arrows indicate regions of RNFL loss. (**E**) RNFL quantification of the region shown in (**D**).

To relate the IOP-induced damage to retinal axons, this study examined the RNFL using OCT. OCT is an imaging technique to record retinal cross-sections in vivo (Figure A2). The analysis of these images is performed using a semi-automatic algorithm, which calculated the thickness of the RNFL calculated in the different regions of the retina, nasal superior (NS), nasal (N), nasal inferior (NI), temporal superior (TS), temporal (T), and temporal inferior (TI) (Figure 3A). RNFL thickness (RNFLT) was examined longitudinally at the indicated time points, with average RNFLT calculated across all regions (Figure 3B). In the IgG OS group, a steady loss of RNFLT was seen longitudinally. This was also evident in the DNM1L OS group, but the overall loss was significantly less compared with the IgG OS group. RNFLT in the contralateral eyes, IgG OD, and DNM1L OD remained approximately identical for the whole study. At week ten, the RNFLT of the IgG OS group was significantly lower at 85 ± 2% compared with the RNFLT of the DNM1L OS group at 97 ± 3% (*p* < 0.001). These relative values referred to the respective baseline examination. The contralateral eyes had RNFLT of 98 ± 2% (IgG OD) and 99 ± 1% (DNM1L). The RNFLT difference between the IgG OS and IgG OD groups was also statistically significant (*p* < 0.001).

The regional RNFLT was analyzed for w10. In the temporal superior region (TS, Figure 3C), the RNFLT of the IgG OS group was 81 ± 7%, significantly lower than the RNFLT of the DNM1L OS group (94 ± 8%, *p* < 0.01) and the IgG OD group (102 ± 5%, *p* < 0.001). In the temporal region (T, Figure 3D), the RNFLT of the IgG OS group had an RNFLT of 82 ± 11%, and the DNM1L OS group had a significantly higher RNFLT of 96 ± 8% (*p* < 0.01). Additionally, a significant different was observed in the RNFLT of the IgG OS group compared with the contralateral eyes (IgG OD, 99 ± 5%, *p* < 0.01). In the temporal inferior region (TI, Figure 3E), the RNFLT of the IgG OS group was 80 ± 14%, whereas the RNFLT in the DNM1L OS group was 102 ± 11%, a significant increase (*p* < 0.01). The RNFLT of the contralateral IgG OD group (97 ± 7%) was also significantly higher than the IgG OS group.

No significant differences were observed in the temporal sectors NS, N, and NI (Figure 3F–H). Therefore, analysis of OCT data showed that application of anti-DNM1L Ab both longitudinally and cross-sectionally markedly attenuated a reduction in the RNFLT. Furthermore, these effects also appeared regionally, as, in the temporal regions, the RNFLT of the DNM1L OS group was significantly higher when compared to the IgG OS group.

The differences between the IgG and DNM1L OS groups within the temporal regions were similar. The difference was approximately 15% in the regions TS and TI. The difference in the TI region was about 20%.

### 2.4. Improvement of the Functional Properties of the Retina by the Application of Anti-DNM1L Ab

**Figure 4:**
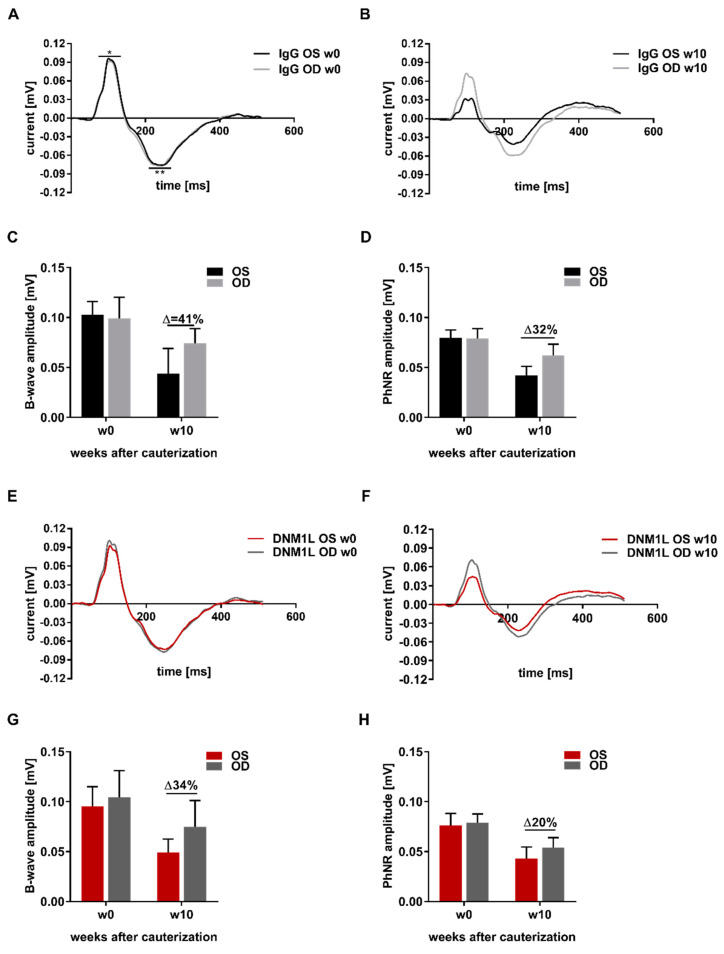
Analysis of retinal functionality using photopic Ganzfeld ERG. To determine retinal functionality, photopic Ganzfeld ERGs of a protocol with increasing light flash intensity were acquired. ERG recordings at baseline (w0, before episcleral vein occlusion, EVO) and at the final examination at w10 after EVO were arithmetically averaged. For the ERG recordings and quantification shown, the flash intensity 1.37 log_10_ cd·s·m^−2^ was used. Thereby, the ERG profile for the IgG group in w0 (**A**) showed no difference between the left (OS) and right (OD) eyes. For quantification, the amplitudes of the profiles, B-wave (*), and photopic negative response (PhNR, **) were used. (**B**) The ERG profile of the IgG group at w10 showed a decrease in B-wave and PhNR amplitudes. (**C**) For quantification in the IgG group, B-wave amplitude (**C**) and PhNR amplitude (**D**) were analyzed. For the DNM1L group, ERG profiles were acquired at time point w0 (**E**) and time point w10 (**F**). B-wave amplitude (**G**) and PhNR amplitude (**H**) were analyzed for w0 and w10. The millivolt decrease was calculated as percent difference (Δ = % change). Number of animals used: *n* = 7 (IgG), *n* = 8 (DNM1L).

A photopic Ganzfeld electroretinogram (ERG) was used to investigate the IOP-induced damage on retinal functionality. The patterns of ERGs of IgG OS and IgG OD were very similar at baseline (w0) concerning B-wave amplitude (*) as well as PhNR amplitude (**) (Figure 4A). The ERG patterns in w10 showed decreased B-wave and PhNR amplitude in the IgG OS (Figure 4B). Quantifying B-wave amplitude (Figure 4C) and PhNR amplitude (Figure 4D) was performed for the indicated time points and showed a 41% decrease in B-wave amplitude in w10 OS compared with OD. The PhNR amplitude in the OS in w10 was 32% lower compared with OD. The ERG pattern of the DNM1L group showed no difference between the OS and OD in w0 (Figure 4E). The ERG pattern of the DNM1L group in w10 showed less pronounced amplitudes in the OS compared with OD (Figure 4F). In w10, the B-wave amplitude was 34% lower in the OS compared with OD (Figure 4G). PhNR amplitude in w10 was 20% lower in the OS compared with OD. In conclusion, the ERG measurements showed that the application of the anti-DNM1L Ab had a more positive effect on the PhNR amplitude, which mainly originated from the RGCs, than on the B-wave amplitude, which mainly originated from bipolar cells.

### 2.5. Application of Anti-DNM1L-Ab Altered DNM1L Phosphorylation and Expression of Apoptosis-Related Proteins

**Figure 5:**
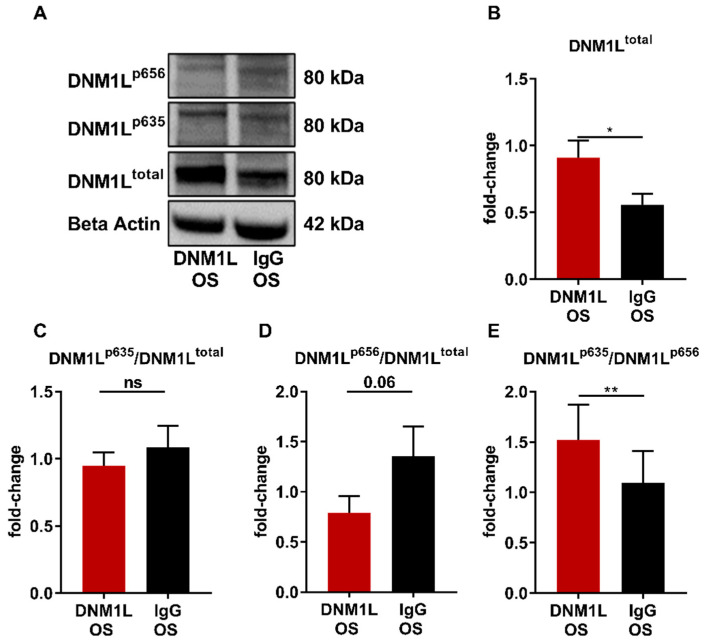
Anti-DNM1L Ab injection alters the phosphorylation of DNM1L. (**A**) Western blot detection of DNM1L, total protein expression (DNM1L^total^), phosphorylation of DNM1L at serine 635 (DNM1L^p635^), phosphorylation of DNM1L as serine 656 (DNM1L^p656^), and housekeeping protein beta-actin. The presented representative western blot is one of six biological replicates. Densitometric analysis of DNM1L^total^ (**B**). All calculated values were normalized to beta-actin. The resulting fold-change was subsequently normalized to the total DNM1L protein expression: (**C**) DNM1L^p635/total^), (**D**) DNM1L^p656/total^. (**E**) The ratio of the DNM1L phosphorylation sites p635 and p656 was calculated (DNM1L^p635^/DNM1L^p656^). All values are given as mean ± SEM calculated from six biological replicates. Statistical analysis was performed using student’s *t*-test. Significance level: * = *p* < 0.05, ** = *p* < 0.01, ns = not significant. Numbers of animals used: *n* = 6 (IgG), *n* = 6 DNM1L.

**Figure 6:**
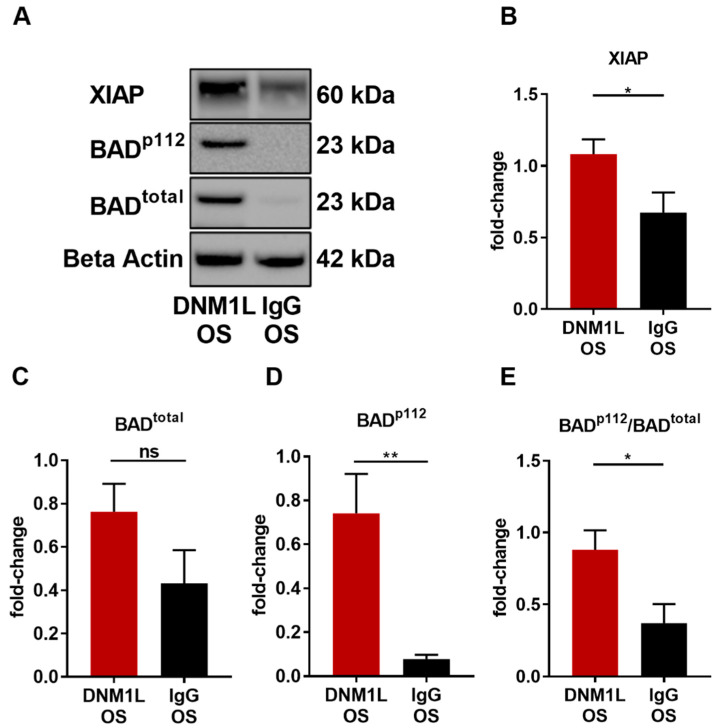
Anti-DNM1L Ab injection reduces proteins involved in mitochondrial apoptosis. (**A**) Western blot detection of XIAP, total protein expression of BAD (BAD^total^), phosphorylation of BAD at serine 112 (BAD^p112^), and housekeeping protein beta-actin. The presented representative western blot is one of six biological replicates. Densitometric analysis of XIAP (**B**), BAD^total^ (**C**), and BAD^p112^ (**D**). All calculated values were normalized to beta-actin. The ratio of BAD^p112^/BAD^total^ was calculated (**E**). All values are given as mean ± SEM calculated from six biological replicates. Statistical analysis was performed using student’s *t*-test. Significance level: * = *p* < 0.05, ** = *p* < 0.01, ns = not significant. Numbers of animals used: *n* = 6 (IgG), *n* = 6 DNM1L.

Western blot analysis was performed to investigate the influence of anti-DNM1L Ab injection on the expression levels of DNM1L protein (Figure 5A) and other apoptosis-related proteins (Figure 6A). Thereby, DNM1L total protein (Figure 5B, DNM1L^total^) exhibited a fold-change of 0.91 ± 0.12 in the DNM1L OS group, which was significantly increased compared with a fold-change of 0.56 ± 0.08 in the IgG OS group (SEM, *p* < 0.05, student’s *t*-test). To conclude DNM1L activity, the fold-changes of phosphorylation at serine 635 (DNM1L^p635^) and serine 656 (DNM1L^p656^) were also examined and normalized to the total protein amount. DNM1L^p635^ showed a fold-change of 0.95 ± 0.10 (Figure 5C, DNM1L^p635/total^) in the DNM1L OS group. The fold-change in the IgG OS group was similar with 1.08 ± 0.16; no significant difference was observed between both groups. For DNM1L^p656^ (Figure 5D, DNM1L^p656/total^), on the other hand, the fold-change in the DNM1L OS group (0.79 ± 0.42) was considerably lower than in the IgG OS group (1.43 ± 0.32, *p* = 0.053). The ratio of phosphorylation for each other (Figure 5E, DNM1L^p635/p656^) was 1.52 ± 0.35 in the DNM1L OS group. The IgG OS group’s ratio was significantly lower at 1.04 ± 0.29 (*p* < 0.01).

To determine the effect of anti-DNM1L Ab injection on apoptosis, the expression levels of X-linked inhibitor of apoptosis (XIAP) and Bcl-2 antagonist of cell death (BAD) were identified, and its phosphorylation site serine 112 (BAD^p112^) was analyzed. XIAP expression was significantly increased in the DNM1L OS group (fold-change: 1.10 ± 0.12) compared with the IgG OS group (fold-change: 0.69 ± 0.15, *p* < 0.05, student’s-test). The BAD total protein fold-change in the DNM1L OS group was 0.76 ± 0.13, and in the IgG OS, 0.43 ± 0.15 (Figure 6B, BAD^total^). However, the phosphorylation site BAD^p112^ was 0.74 ± 0.18 in the DNM1L OS, and the IgG OS was 0.08 ± 0.02, which significantly decreased (Figure 6D, *p* < 0.05). The pBAD/BAD ratio (Figure 6E, BAD^p112/total^) in the DNM1L OS group was 0.88 ± 0.13, and in the IgG OS group, it was 0.37 ± 0.13.

### 2.6. Proteomic Analysis of Retinal Tissues to Evaluate Alterations upon Anti-DNM1L Ab Application

**Figure 7:**
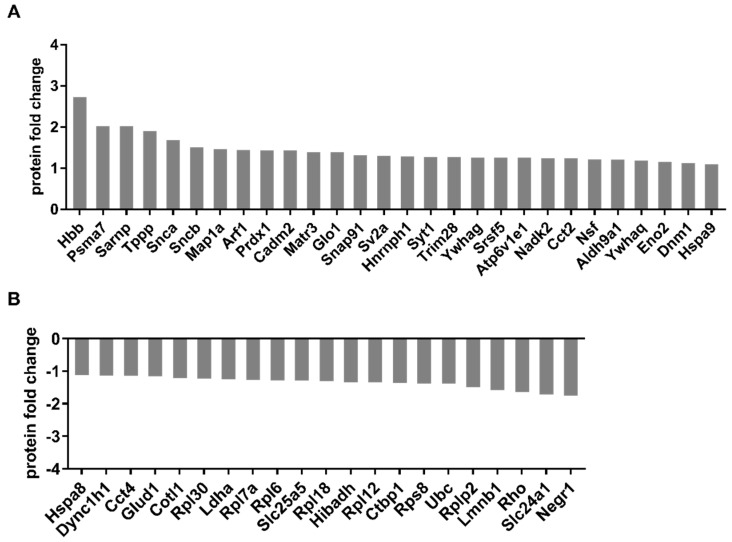
Illustration of the up- and down-regulation of significantly altered protein expression identified by mass spectrometric analysis. After sacrifice of the animals at the end of the study, the proteins were isolated from retinal tissue and tryptically digested. The resulting peptides were purified and analyzed by MS. Identification, quantification and normalization of the peptides was performed using MaxQuant. For statistical analysis, a Student’s *t*-test was used with a significance level *p* < 0.05. The graph shows the up—(**A**) and down-regulated (**B**) proteins in the antibody-treated group compared to the control group. Numbers of animals used: *n* = 7 (IgG), *n* = 8 DNM1L.

To analyze the protein changes induced by anti-DNM1L Ab injection, mass spectrometric (MS) analysis of the whole retinal tissue was performed. A total of 968 proteins were identified in both experimental groups (IgG and DNML1 OS). Of these, 28 were significantly up-regulated, and 21 were significantly down-regulated (student’s *t*-test, Figure 7).

**Figure 8:**
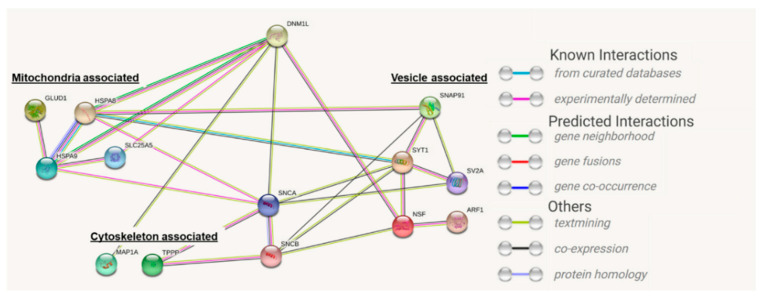
STRING protein interaction network. Analysis of the signaling pathway altered by the anti-DNM1L Ab injection. Search tool for the retrieval of interacting proteins (STRING) shows the signaling pathways of the most significantly changed proteins using the medium confidence score (0.4). The target protein of the study, DNM1L, was added manually to the network.

Clusters of significantly changed proteins can be identified, which might be related to neuronal activity or mitochondrial dynamics. To identify the association of these proteins with DNM1L, a STRING protein–protein interaction network analysis was performed (Figure 8).

The network showed significantly altered proteins (*p* < 0.05, student’s *t*-test) identified in the MS analysis. The target protein of this study, DNM1L, was not identified as significantly different in the present MS analysis and was manually added to determine possible interactions with the significantly changed marker protein.

The interaction network demonstrated that DNM1L interacts directly with the proteins alpha-synuclein (SCNA), N-ethylmaleimide sensitive fusion proteins (NSF), heat shock proteins 8A and 9A (HSP8A, HSP9A), ADP/ATP translocase 2 (SLC25A5, ANT2), and microtubule-associated protein 1A (MAP1A). These direct protein interactors are connected to further interaction subnetworks. According to their association or function within the cell, they were categorized as mitochondrial-associated, cytoskeleton-associated, or involved in vesicle trafficking (Table 1).

## 3. Discussion

Mitochondrial dysfunction is a cause of lower metabolic activity in glaucoma patients, which in turn represents stress conditions for retinal cells, especially retinal ganglion cells (RGCs), resulting in cell death. These processes are similar in glaucoma patients and animal models [23]. The animal model used in this study induced chronic IOP elevation in Spraque Dawley rats by occluding three of four episcleral veins (EVO) of the OS to mimic glaucoma-like conditions [24]. EVO resulted in a significantly increased IOP, which remained stable until the end of the study and had similar levels as in previous studies of our group [25,26]. Additionally, the significant loss of RGCs and the magnitude of RNFLT thinning in the IgG OS group were within the expected range [25,26,27]. Considering the regional RGC density (Figure 2), it was noticeable that the protective effect of anti-DNM1L Ab mostly occurred in the central and mid-peripheral regions. As the pressure sensitivity of RGCs increases towards the periphery, RGC survival in our study could be caused by RGCs, which have not fully activated the apoptosis signaling pathways yet [28,29,30]. Additionally, the viable RGCs might be capable of regeneration instead of going into apoptosis [31,32].

Regarding retinal functionality, chronic IOP elevation in ERG patterns showed a noticeable decrease in B-wave amplitude in a similar range compared to another study [27]. However, it points out that not only inner but also outer retinal layers were affected by chronic IOP elevation. This might be caused by photoreceptor cells affected by IOP, as shown by Nork et al. (2000) [33]. A lower PhNR amplitude was expected since it mainly originated from the RGCs, which were remarkably decreased due to the IOP elevation. Injection of the anti-DNM1L Abs mitigated the reduction in PhNR amplitude compared to the control IgG, probably due to the neuroprotection of RGCs in the antibody-treated eyes.

Nevertheless, it should be noted that the preservation of structure does not necessarily imply a positive effect on functionality. Indeed, ERG cannot determine from which RGC the electrophysiological response precisely originated. Nevertheless, it has previously been shown that targeting mitochondria, which enhances energy metabolism in RGCs, can positively affect retinal functionality, especially the PhNR [34,35]. In summary, the present study demonstrated that anti-DNM1L Ab injection induced positive neuroprotective effects at both physical and physiological levels.

Mitochondrial dynamics, especially fission and fusion, are indispensable for mitochondrial distribution and homeostasis [36,37]. The DNM1L protein is crucially involved in this process, and its function is determined by various post-translational modifications (PTMs) such as phosphorylation, ubiquitylation, S-nitrosylation, and sumoylation. These modifications collectively regulate the mitochondrial fission rate [38,39]. The best-described phosphorylation sites of DNM1L are Ser635 and Ser656. Phosphorylation of Ser635 stimulates DRP1 activity leading to increased mitochondrial fission, while phosphorylation of Ser656 inhibits fission [38,40]. Protein kinase A (PKA)-induced phosphorylation of Ser656 reduces the GTPase activity of DNM1L and promotes DNM1L dissociation from the outer mitochondrial membrane. Dephosphorylation of Ser656 occurs via Ca^2+^-activated calcineurin triggering fission [41,42].

To investigate the molecular effects of anti-DNM1L-Ab injection, total DNM1L protein expression and the phosphorylation status of Ser635 (DNM1L^p635^) and Ser656 (DNM1L^p656^) were analyzed. Western blot analysis of retinal tissues revealed increased DNM1L^total^ abundance in the DNM1L OS group (Figure 5B), suggesting increased mitochondrial dynamics and altered localization.

The function of DNM1L is versatile and is not only based on the total protein expression but is strongly influenced by various PTMs. The ratio of the phosphorylation Ser635 to Ser656 indicates higher mitochondrial fission. In turn, more DNM1L is required for fission. This would correspond to a kind of positive forward loop and could be an explanation for increased DNM1L levels. Therefore, the DNM1L total protein expression is an indicator; the phosphorylation ratio to each other (normalized to the total protein in each case) is even more decisive. However, these are considerations, and it should bear in mind that the data shown show a high standard deviation. Therefore, we do not want to overinterpret the data but consider them as indications that need to be addressed in future studies in more detail.

Studies demonstrated that DNM1L could impact the localization of mitochondria or vesicles. Mitochondria are transported to sites of increased energy demand in response to DNM1L expression, thereby regulating synapse formation and axon branching [16,43]. However, mitochondrial dynamics depend on several factors, such as PTMs. In this regard, the DNM1L^p635/total^ expression level was similar in both study groups (Figure 5C).

In contrast, the phosphorylation site DNM1L^p656/total^ expression level was considerably lower in the DNM1L OS group compared to the IgG OS (Figure 5D). Decreased DNM1L^p656^ level might indicate increased mitochondrial fission [44]. Since the two phosphorylation sites synergistically regulate DNM1L activity, it is not sufficient to consider the expression level separately but to compare the ratios of both phosphorylation sites in combination (Figure 5E, DNM1L^p635/p656^). This ratio was moderately but significantly increased in the DNM1L OS group compared with IgG OS, indicating increased mitochondrial fission.

However, the DNM1L function is determined by several pathways, not only by PTMs. Therefore, it was necessary to analyze the changes in protein expressions in retinal tissues to discuss possible molecular mechanisms and effects in retinal cells treated with anti-DNM1L. Quantitative mass spectrometric (MS) analysis and subsequent STRING pathway analysis revealed that some significantly altered proteins could be subdivided into the three protein clusters vesicle trafficking, cytoskeleton-associated, and mitochondrion-associated signaling. The latter is of particular interest due to the mitochondrial association of DNM1L and includes the proteins HSPA9, ANT2, and GLUD1 (Table 1).

In our study, heat shock protein A9 (HSPA9) was significantly increased in the DNM1L OS group compared with the IgG OS. HSPA9 (Mortalin) is a member of the HSP70 family, which can be inducibly expressed as a direct physiological response to stress [44].

Tanaka et al. (1995) showed that mortalin is a mitochondrial chaperone protein recently described as a sensor of neuronal stress and involved in protein folding quality control [45,46]. It is also down-regulated in the neurons of patients with neurodegenerative diseases, such as AD [47] or PD [48,49]. In an experimental autoimmune encephalomyelitis (EAE) model, mortalin ameliorated mitochondrial dysfunction and visual loss in a gene therapy approach [50,51,52]. Studies have shown that mortalin expression levels in neurons directly correlate with mitochondrial stress control, which can also be the occurrence of ROS, as it has already been described for non-neuronal cells and primary cultures of cortical neurons [50,53]. The increased expression of HSPA9 discussed previously might indicate increased contact sites between mitochondria and ER, resulting in increased Ca^2+^ influx into mitochondria [54,55]. However, an excessive influx of Ca^2+^ could lead to apoptosis [56,57]. Conversely, the adenine nucleoid translocase 2 (ANT2) expression in the DNM1L OS group decreased. This protein is an antiporter by exchanging ADP/ATP within the mitochondrial permeability transition pore complex (MPTP) between the mitochondrial matrix and the cytoplasm [58,59]. Therefore, ANT2 plays a key role in maintaining mitochondrial membrane potential, regulating the ADP/ATP ratio during oxidative phosphorylation [60,61]. ANT2 facilitates mitochondrial membrane uncoupling when acetylated by SIRT4, leading to apoptosis [58,59]. Loss of ANT1 and ANT2 reduces desensitized Ca^2+^-induced MPTP opening in mitochondria [62,63]. Thus, ANT2 inhibition leads to inhibition of cytochrome c efflux from the inner mitochondrial membrane, which recruits in the suppression of apoptosis [60,61]. However, at high Ca^2+^ concentrations, MPTP could still open [64]. That inhibition of uncoupling proteins in the retina can be protective and has been demonstrated in cell culture experiments [65,66]. The decreased expression of ANT2 in the DNM1L OS group could reduce apoptosis, which follows the previously discussed increased expression levels of BAD and XIAP.

Pathophysiological processes such as oxidative stress or glutamate excitotoxicity lead to increased apoptosis of RGCs in glaucoma [9,10,12]. Therefore, it is not surprising that glutamate dehydrogenase (GLUD1) is up-regulated by high IOP and is mainly located in the retinal tissues [67]. In our study, GLUD1 was significantly less abundant in the DNM1L OS group compared to the IgG OS. In glutamate metabolism, GLUD1 has been associated with neurodegenerative properties in neuronal tissues [68,69], as GLUD1 can stimulate the Krebs cycle, resulting in increased oxidative phosphorylation and ROS production, in turn, is detrimental to RGCs [70]. Therefore, the lower abundance of GLUD1 in the DNM1L OS group of our study suggested reduced cellular stress by ROS due to anti-DNM1L Ab injection.

In summary, our study demonstrated that anti-DNM1L-Ab injection affected DNM1L expression and phosphorylation status at Ser635 and Ser656. In addition, mitochondrial proteins were found significantly altered in the DNM1L OS group, which could indicate altered mitochondrial biogenesis. However, these proteins do not appear to interact with DNM1L directly but rather result from altered DNM1L expression or phosphorylation state.

DNM1L regulates not only the mitochondrial division but also mitochondrial distribution. In this process, DNM1L interacts with the cytoskeleton or cytoskeleton-associated proteins, including MAP1A, TPPP, and COTL1, which were significantly differentially expressed between both study groups. MAP1A and the TPPPs were more abundant in the DNM1L OS group compared to IgG OS, indicating an increased MT polymerization, stabilization, and extension of the MT network [71]. Elevated IOP seems to decrease the MAP1A amount and, thereby, the number of axonal cross-bridges, leading to microtubule instability and abnormal organelle transportation in axon plasma [72]. TPPPs show extensive MT bundling and polymerization abilities critical for axon formation, extension, and guidance at nerve terminals, as demonstrated in retinae of the model organisms drosophila, zebrafish, mouse, and human [73,74,75,76,77,78,79]. A study investigating AD showed TPPP down-regulation [80]. Our study showed that the MT-associated proteins, MAP1A and TPPP, were significantly up-regulated, suggesting increased MT stability. An actin-associated protein, coactosin-like protein 1 (COTL1), was also significantly less abundant in the DNM1L OS group (Figure 7). COTL1, as an actin depolymerization factor (ADF) and member of the cofilin family, plays a crucial role in binding to F-actin and thus preventing polymerization [81]. COTL1 overexpression showed disturbed neuronal morphology and exhibited AD-like neuropathology [82,83]. In a study with POAG patients, COTL1 was up-regulated in aqueous humor [84]. Therefore, the low abundance of COTL1 suggested continued polymerization and stabilization of the actin cytoskeleton, which, together with MT cytoskeleton stabilization, may be part of a neuroprotective mechanism, as shown in previous studies [26,85].

In this context, increased DNM1L abundance appeared to be a crucial factor, as DNM1L is required for proper mitochondrial distribution and mobilization of synaptic vesicles [86]. Increased vesicle trafficking and altered mitochondrial distribution could occur anterograde towards energy-demanding regions [87]. Decreased cytoplasmic dynein 1 heavy chain 1 (DYNC1H1, Figure 7) abundance in the DNM1L OS group indicated reduced retrograde cargo transportation towards the soma in the DNM1L OS group [37,88,89,90]. Transportation of mitochondria or vesicles along microtubules in the direction of the pre-synapse occurs via kinesin heavy chain (KHC) proteins and milton [91]. DNM1L can bind kinesin light chain (KLC) proteins, which are KHC blocking proteins so that the interaction of KHC with cargo can occur [92]. The increased DNM1L expression in the antibody-treated group might promote cargo transportation towards synapses, e.g., mitochondria or synaptic vesicles. This hypothesis could be supported by the significantly altered proteins associated with vesicle trafficking, including SNAP91, NSF, NEGR1, SYT1, and SV2A.

SNAP91, NSF, SYT1, and SV2A were significantly more abundant in the DNM1L OS group compared with IgG OS. These proteins are involved in the distribution and clustering of synaptic vesicle cargo [93,94,95]. In this context, SNAP91 maintains the distribution of proteins in the synapse and vesicle at nerve termini and regulates exocytosis [96,97]. The manipulation of SNAP91, such as mutations or loss of function, is associated with abnormal vesicles or perturbed neurotransmitter exocytosis and is also involved in PD [98,99,100]. The NSF protein provides the necessary energy for vesicle dissociation as an AAA ATPase. However, it has also been shown that NSF and neuronal growth factor 1 (NEGR1) may also regulate vesicle fusion and recycling [101,102]. Overexpression of NEGR1 resulted in reduced synapse formation and reduction in SNARE proteins, which play an important role in vesicle formation [102]. NSF was down-regulated in a study with PD patients, indicating reduced vesicle trafficking, the main feature of the disease [103,104]. Neuroprotective mechanisms found in cell culture and experimental mouse models for PD were associated with the up-regulation of Snap91 and NSF [105]. NEGR1 down-regulation in the DNM1L OS group suggested improved synapse formation and increased abundance of SNARE proteins such as SNAP91, which might be underlined by increased NSF and AAA ATPase abundance providing the required energy.

Down-regulation of ATP6V1A, which also plays a critical role in vesicular transport and oxidative phosphorylation, has been implicated in AD [106]. In an experimental autophagy model, down-regulation of ATP6V1A led to neurodegeneration, defective lysosomal function, and autophagy arrest, which increased the vulnerability of RGCs [107]. Since ATP6V1A expression was up-regulated in the DNM1L OS group in our study, this seems to play a neuroprotective role.

Exocytosis of synaptic vesicles, containing neurotransmitters, occurs by SV2A and SV2B, among others [108,109,110,111], maintaining neuronal communication between pre- and post-synapses [112]. SV2A was up-regulated in the DNM1L OS group compared to the IgG OS in our study. Low expression or dysfunction of SV2A has been shown to initiate neurodegenerative processes, including neuronal apoptosis, axon damage, and synapse loss, in AD and ocular hypertension [113,114]. Therefore, increased SV2A abundance may indicate increased exocytosis and thus enhanced neuronal activity.

Although the presented study showed a new approach for an IOP independent glaucoma therapy, some study limitations should be pointed out. Different animal models may emphasize signal transduction pathways differently. Therefore, a single model cannot reflect all features of a multifactorial disease such as glaucoma. Because of limited tissue amounts, this study focused on analyzing the target protein, DNM1L, certain phosphorylation, and mitochondrial apoptosis-related proteins. For the analysis of proteomic changes in retinal tissues with minimal tissue usage, mass spectrometric analysis was performed. Our study demonstrated that EVO altered DNM1L phosphorylation status at Ser635 and Ser656, which indicates a higher fusion rate than the division rate in the IgG OS group. Abnormally high fusion of mitochondria results in hyper elongated mitochondria and has been described as a feature of senescent cells [115,116]. The senescence of retinal cells after stress induction has been previously reported as a reversible state to prevent death [31]. Based on the data of our study, we propose that anti-DNM1L Ab injection altered the phosphorylation status, possibly by blocking the phosphorylation site at Ser656, such that cells could return from a senescent state to a biologically active state. This, in turn, could trigger increased mitochondrial division, leading to increased synthesis of DNM1L, as shown by western blot.

Consequently, a stabilization of the cytoskeleton could occur since, by mass spectrometry, cytoskeleton stabilizing proteins were found to be significantly increased in the DNM1L OS group and could lead to an increased survival of RGCs. In the study of mitochondrial dynamics, fission and fusion are crucial processes as well as biogenesis, i.e., the synthesis of mitochondrial proteins. Therefore, it is also important to see the proteomic changes concerning the total mitochondrial content.

Interestingly, we detected by mass spectrometry proteins of the mitochondrial cytochrome c reductase complex (cytochrome b-c1 complex subunit 1, mitochondrial (UQCRC2), cytochrome b-c1 complex subunit 2, mitochondrial (UQCRC2)) which were not significantly altered in the IgG OS or DNM1L OS, suggesting that the total mitochondrial content seems unaltered by the treatment. The hypotheses put forward in this manuscript are based on the functional properties of the identified proteins as published in other studies and disease models such as PD or Alzheimer’s disease. For concrete evidence of altered mitochondrial dynamics and mitochondrial distribution further studies need to be conducted with this focus, for example, by electron microscopy. Nevertheless, these results highlight the protective effect of anti-DNM1L-Ab injection and provide clues to the molecular background worth pursuing further, opening new options for potential glaucoma therapy.

## 4. Materials and Methods

### 4.1. Animals

All experiments in this study were performed according to the Association for Research in Vision and Ophthalmology (ARVO) guidelines. The methods used were reviewed and approved by the National Investigation Office of the State of Rhineland-Palatinate in Koblenz, Germany, concerning the experimental project’s legal requirements and ethical acceptability (23 177-07/G15-1-053). Female Spraque Dawley rats aged seven weeks and about 190 g body weight were used for the study.

### 4.2. Experimental Design

All animals used in this study were kept under controlled temperature and humidity. Meanwhile, the animals always had ad libitum access to water and chow. Each animal received baseline examinations of intraocular pressure (IOP) by rebound tonometry, retinal nerve fiber layer thickness (RNFLT) by optical coherence tomography (OCT), and retinal functionality by electroretinogram (ERG) before the start of IOP elevation (week 0, w0). These assessments were performed longitudinally at week 3 (w3), week 6 (w6), week 8 (w8), and week 10 (w10). Each group received manipulations exclusively on the left eyes (OS). The right eyes (OD) of the animals remained completely untreated. IOP elevation was induced by episcleral vein occlusion (EVO) in the left eye (OS) in the week after baseline examination; the right eye (OD) remained untreated and served as an internal control. Intravitreal injection (IVI) of anti-DNM1L antibody (anti-DNM1L Ab) or the IgG control antibody into the OS was performed after stable IOP elevation in w3 after EVO. The animals were divided into two groups according to the substance applied. One group (IgG OS, *n* = 7) received a control antibody (Rabbit IgG isotype control, Antibodies Online, ABIN398653) with unknown specificity. The other group (DNM1L OS, *n* = 8) received an anti-DNM1L Ab (702782, Invitrogen/Thermo Fisher Scientific, Waltham, MA, USA). The contralateral OD eyes were not manipulated with EVO or IVI. These eyes were named after the substance was injected into the OS of the respective animals, IgG OD or DNM1L OD. At ten weeks post-EVO, animals were sacrificed by carbon dioxide overdose.

### 4.3. Intraocular Pressure (IOP) Monitoring by Rebound Tonometry

In this study, IOP was measured with a TonoLab rebound tonometer specially designed for rodents (iCare, Espoo, Finland). The animal is held horizontally as possible for a correct measurement. The tonometer probe touched the corneal surface at approximately a right angle. Ten consecutive correctly measured values were recorded for calculation of the average value. Outliers caused by the device or the animal’s movement were excluded and not recorded. Measurements were taken weekly between 8 a.m. and 12 p.m.

### 4.4. Chronic IOP Elevation by Episcleral Vein Occlusion (EVO)

Animals were anesthetized systemically with a mixture consisting of 0.25 mg/kg medetomidine (1 mg/mL Dorbene vet., Zoetis Deutschland GmbH, Berlin, Germany) and 0.7 mg/kg ketamine (Ketamine Inresa, 50 mg/mL, Inresa Arzneimittel GmbH, Freiburg, Germany) before surgery. Additionally, the eye was topically anesthetized with one or two drops of oxybuprocaine hydrochloride (Novesine^®^ 0.4% eye drops, OmniVision^®^, OmniVision GmbH, Puchheim, Germany). EVO was performed on the OS only, as described by Shareef et al. 1995 [117]. Three of the four episcleral veins were thermally cauterized after they were exposed. Finally, the incision was closed with surgical knots, and both corneas were protected with bepanthene ointment.

### 4.5. Intravitreal Injection (IVI) of Antibodies

In this study, IVI of the antibodies was performed into the OS after stable IOP elevation immediately after the regular in vivo assays in w3. 25 µg of the anti-DNM1L antibody or the IgG control antibody was injected at a volume of 3.5 µL using a 10 µL Hamilton syringe [118,119].

### 4.6. RNFL Thickness Measurement by Optical Coherence Tomography (OCT)

Short-term anesthesia of the animals for the examination was performed using medetomidine. Anesthesia was antagonized by atipamezole after the examination. Before the examination, the pupil was dilated using topical administration of tropicamide (Mydriaticum, Pharma Stullen, Stullen, Germany). The eye’s closing reflex was anesthetized using topical Novesine application. By using spectral-domain OCT, RNFL thickness was determined in this study. A circular B-scan was acquired around the optic disc with a 12° diameter. The B-scan was analyzed using Heidelberg Eye Explorer software as in previous studies [26]. RNFL limits were determined semiautomatically (Figure A2) [26]. The software calculated the average RNFL thickness in different sectors: temporal superior (TS), temporal (T), temporal inferior (TI), nasal superior (NS), nasal (N), and nasal inferior (NI). In addition, the software calculated the average RNFL thickness over the entire scan. The timing of the scans was done as described above in w0 as the baseline and in w3, w6, w8, and w10 after EVO.

### 4.7. Photopic Ganzfeld Electroretinogram (ERG)

Anesthesia was performed for OCT examination and pupil dilation using Mydriaticum and subsequent topical anesthesia using Novesine. The animals were prepared for ERG examination as described previously. The photopic Ganzfeld ERG was prepared with the RETI system (Roland Consult, Brandenburg, Germany) [27]. A green background light with 40 cd∙s∙m^−2^ was used to mimic photopic conditions during the recordings. The intensity sequence protocol used has been applied in previous studies [27]. The white light stimuli included intensities −0.15, 0.23, 0.61, 0.99, and 1.37 log_10_ cd∙s∙m^−2^. 25 ERGs were recorded for each intensity, and the mean value was used for subsequent analysis; artifacts were automatically excluded during recording. The recording time of each ERG was 512 ms, and the frequency of successive stimuli was 0.33 Hz.

### 4.8. Immunofluorescence Staining of Retinal Ganglion Cells of Retinal Flatmounts

After the study, the animals were sacrificed, and the eyes were enucleated. Following this, the retina was dissected as previously described [26]. Indirect immunofluorescence staining was used to stain RGCs with the retinal ganglion cell marker brain-specific homeobox/POU domain protein 3 A (Brn3a). For this purpose, the primary goat anti-Brn3a polyclonal antibody (1:125, C-20, 31,984 Santa Cruz Biotechnology, Santa Cruz, CA, USA) and the secondary anti-goat antibody coupled with AlexaFluor568 (1:400, #A-11057, Invitrogen, Carlsbad, CA, USA) were used according to a previously established protocol [26]. The retina was transferred to a glass slide and covered with Vectashield mounting medium (VECTASHIELD Antifade Mounting Medium, H-1000, Vector Laboratories, Burlingame, CA, USA). Finally, it was capped with a coverslip. Images of Brn3a positive cells (Brn3a^+^ cells) were acquired using the Eclipse TS 100 fluorescence microscope (Nikon, Yurakucho, Tokyo, Japan) with a DS-Fi1-U2 digital microscope camera (Pixel pitch 3.5 µm, Nikon) and an ELWD 20×/0.45 S Plan Flour Ph1 ADM objective (Nikon). The microscope used the NIS Elements recording software (Nikon, version 4.10 64-bit). The images have a size of 0.142 mm^2^. Nine images of each retinal quarter were acquired to evaluate Brn3a^+^ cells. The quarter was divided into central, mid-peripheral, and peripheral areas. Three recordings were made for each area (Figure A1). The RGC number was determined using a semiautomated macro of ImageJ software (http://rsb.info.nih.gov/ij/, NIH, Bethesda, MD, USA). The macro included four steps: (1) convert to 8-bit, (2) subtract background, rolling ball radius 120, (3) set auto threshold automatically, (4) run “nucleus counter” with smallest 400 and largest 7000. Analysis was performed in a blinded manner.

### 4.9. Protein Extraction

Mass spectrometry and western blot analysis were performed to analyze the protein composition and identify altered protein abundances in the retinal tissue. For this purpose, proteins were isolated from retinal tissue, as described in previous publications [118,119]. The Tissue Protein Extraction Reagent buffer (T-PER, Thermo Fisher Scientific, Rockford, IL, USA) was used. According to the manufacturer’s instructions, in deviation from the published protocol, a protease inhibitor cocktail (cOmplete™ EDTA-free Protease Inhibitor Cocktail Tablets, 11697498001, Basel, Switzerland Merck, Darmstadt, Germany) and a phosphatase inhibitor (PhosSTOP™, 04 906 845 001, Roche), were added to the T-PER buffer in this study. This customized buffer was used to homogenize the retina, followed by buffer exchange from T-PER buffer to LC–MS grade water. Protein concentration was then determined using a BCA assay kit (Thermo Fisher Scientific, Rockford, IL, USA) and analyzed with a Multiscan Ascent photometer (Thermo Fisher Scientific, Rockford, IL, USA) at 570 nm.

### 4.10. Discovery Proteomics

For MS analysis, 10 µg of retinal protein was used and evaporated to dryness in the SpeedVac (Eppendorf, Darmstadt, Germany) for 30 min at 30 °C. The proteins were then digested in-solution by trypsin. Prior to MS analysis, peptide purification was performed via SOLAµ^TM^ spin plates (Thermo Fisher Scientific, Rockford, IL, USA) as described in previous publications [120]. The liquid chromatography–mass spectrometry (LC–MS) measurements were performed with a hybrid linear ion trap Orbitrap MS system (LTQ Orbitrap XL, Thermo Fisher Scientific, Rockford, IL, USA) coupled online to the EASY-nLC 1200 system (Thermo Fisher Scientific, Rockford, IL, USA). Purified and tryptic digested samples were dissolved in 80 µL of a 0.1% formic acid (FA), and 2 µL of each sample (0.125 µg/µL) was injected into the system for each run. Solvent A consisted of 0.1% FA in water, and solvent B consisted of 0.1% FA in 80% ACN. Peptides were separated using a PepMap C_18_ column system (75 µm × 500 mm; Thermo Fisher Scientific, Rockford, IL, USA). Peptide elution was performed in a gradient over 200 min: 5–30% B (0–160 min), 30–100% B (160–180 min), and 100% B (180–200 min). As described in previous publications, the LTQ Orbitrap operated in positive ionization mode and data-dependent acquisition (DDA) mode [120]. The automatic gain control was set to 1 × 10^6^ ions. For internal calibration, the lock mass was set to 445.120025 *m*/*z* (poly-dimethyl cyclosiloxane). The dynamic exclusion mode was enabled with the following settings: repeat count = 1, repeat duration = 30 s, exclusion list size = 100, exclusion duration = 300 s, and exclusion mass width = ±20 ppm. The five most intense precursor ions were selected for collision-induced dissociation (CID) fragmentation in the ion trap with a normalized collision energy of 35%. Raw LC–MS data were analyzed using MaxQuant v. 1.6.17 (Max Planck Institute for Biochemistry, Martinsried, Germany) bioinformatics software for protein identification and quantification [121]. Tandem MS spectra were searched against the SwissProt database with the taxonomy *Rattus norvegicus* (date: 7 July 2021, entries: 8131 sequences). The settings were chosen as previously described. All protein identifications were filtered with a false discovery rate (FDR) < 1% (Supplementary Table S1).

### 4.11. Analysis and Bioinformatics of MS Data

Perseus software, version 1.6.15.0 (Max Planck Institute of Biochemistry, Martinsried, Germany), was used to statistically analyze the MaxQuant-specific output data. At first, contaminants, reversed hits, and protein hits only ‘identified by size’ were excluded from the analysis. Then, the LFQ intensities of the proteins were log_2_ transformed and needed to be detected in at least all biological replicates of one study group (IgG OS: *n* = 7 or DNM1L OS: *n* = 8) before further processing. In addition, protein identifications with at least two unique peptides were accepted for quantitative analysis. Missing intensity values were imputed by random numbers received from the normal distribution (width: 0.3, downshift: 1.8, Supplementary Table S2). The abundances of the identified proteins were plotted in a volcano plot (Supplementary Figure S1). A two-tailed Student’s *t*-test was used to determine statistically significant altered proteins between both experimental groups (IgG OS and DNML1 OS). Protein changes with *p* values < 0.05 were defined as statistically significant (Supplementary Table S3). The mass spectrometry proteomics data have been deposited to the ProteomeXchange Consortium via the PRIDE partner [122] repository with the dataset identifier PXD031987.

### 4.12. Western Blot

50 µg of retinal protein per lane prepared for western blot analysis as described in protein extraction was separated on 4–12% SDS gels (NuPAGE™ 4–12%, Bis-Tris, Invitrogen™, Fisher Scientific, Schwerte, Germany, 10472322) using MES buffer (150 V). Proteins were transferred to a nitrocellulose membrane (Amersham™ Protran™ 0.2 µm Nitrocellulose Blotting Membrane, Catalogue No10600001, GE Healthcare) by tank blot using a standard Towbin buffer [123]. Blotting quality was checked using Ponceau S total protein staining. The membrane was decolorized with Tris-buffered saline (TBS) and subsequently blocked with 5% milk (Difco™ Skim Milk, BD Franklin Lakes, NJ, USA) in TBS-T for one hour. Overnight incubation of the membrane with the primary antibodies, listed in Table 2, was performed at 4 °C. Corresponding secondary antibodies coupled with horseradish peroxidase were incubated for one hour at room temperature. SignalFire™ ECL Reagent (#6883, Cell Signaling Technology^®^, Danvers, MA, USA) was used to develop the membranes according to the manufacturer’s instructions. Detection was performed using the Fluor Chem E system (ProteinSimple, San Jose, CA, USA). For multiple incubations of the membranes, they were stripped with a stripping buffer (0.2M NaOH) for 30 min at room temperature after detection. Subsequently, the membrane was washed several times with TBS. After that, the membrane was ready for further incubation. The densitometric analysis was performed using ImageJ (http://rsb.info.nih.gov/ij/, accessed on 7 July 2022), and the band densities were normalized to the band densities of beta-actin.

### 4.13. Protein–Protein Interaction Network Development

The Student’s *t*-test was used to statistically determine the altered proteins (significance level: *p* < 0.05), comparing the DNM1L and IgG OS groups. Several altered proteins were used to generate a STRING protein interaction network. DNM1L was added manually to relate the target protein to the significantly altered proteins.

### 4.14. Statistical Analysis and Image Processing

Data for this study were expressed as mean ± standard deviation (SD) unless otherwise stated. Statistical analysis was performed using Statistica version 13 software (Dell Inc. Round Rock, TX, USA). Different experimental groups were analyzed using a one-way analysis of variance (one-way ANOVA) followed by a Tukey’s honest significant difference (HSD) post hoc test to perform multiple group comparisons with unequal group sizes. IOP data were statistically analyzed using a two-tailed Student’s *t*-test. Graphs were generated using GraphPad Prism software (GraphPad Software Inc., San Diego, CA, USA).

### 4.15. Ethical Approval

The national investigation office approved all experimental protocols in Koblenz, Germany (23-177-07/G 15-1-053. The methods were carried out following the relevant guidelines and regulations.

## Figures and Tables

**Table 1:** MS identified proteins, categorized by subcellular location or association.

**MS Identified Proteins, Which Are Located in Mitochondria.**		
**Protein Name**	**Protein Name Abbreviated**	**Regulation in the DNM1L OS Group**
Glutamate dehydrogenase 1 ADP/ATP translocase 2 mitochondrial stress-70 protein, mitochondrial	GLUD1 SLC25A5, ANT2 HSPA9	Down Down Up
**MS Identified Proteins Associated with the Cytoskeleton.**		
**Protein name**	**Protein name abbreviated**	**Regulation in the DNM1L OS Group**
Neuronal growth factor 1 Coactosin-like protein 1 Tubulin polymerization promoting protein Microtubule-associated protein 1A	NEGR1 COTL1 TPPP MAP1A	Down Down Up Up
**MS Identified Proteins Associated with Vesicle Traffic.**		
**Protein name**	**Protein name abbreviated**	**Regulation in the DNM1L OS Group**
Clathrin coat assembly protein AP180	SNAP91	Up
Vesicle fusing ATPase	NSF	Up
V-type proton ATPase subunit	ATP6V1E1	Up
ADP-ribosylation factor 1	ARF1	Up
Synaptogamin-1	SYT 1	Up
Synaptic vesicle glycoprotein 2A	SV2A	Up
alpha-Synuclein	SCNA	Up
beta-Synuclein	SCNB	Up

**Table 2:** List of antibodies used for western blot. The antibodies were diluted, if not stated differently, in 5% BSA and 0.1% NaN3 in TBS-T. Abbreviations: rb = rabbit, ms = mouse. * The phosphorylation sites in the human DNM1L are at positions Ser616 and Ser637. Due to a 100% sequence homology in the BLAST comparison of human with rat DNM1L, we assume that the antibody used also detect the rat-specific phosphorylation sites at Ser635 (Ser616, human) or Ser656 (Ser637, human). Since rat tissue was used in this manuscript, we termed the DNM1L phosphorylation sites according to the rat-specific positions.

Antigen	Species	Clonality	Manufacturer	Order No.	Application
Bad	rb	polyclonal	Cell Signaling	9292	1:1000
Bad, phospho (Ser112)	rb	polyclonal	Cell Signaling	9291	1:1000
Beta-actin	ms	monoclonal	Antibodies Online	ABIN3020544	1:5000 (5% milk in TBS-T)
DNM1L	rb	monoclonal	Thermo Fisher Scientific	702782	1:250
DNM1L (DRP1), phospho Ser616 *	rb	polyclonal	Dianova	AF8470	1:1000
DNM1L (DRP1), phospho Ser637 *	rb	polyclonal	Origene	AP55855PU-S	1:1000
XIAP	rb	polyclonal	Cell Signaling	2042	1:1000
Anti-rabbit HRP	gt	n/a	Cell Signaling	7074	1:10,000 (5% milk in TBS-T)
Anti-mouse HRP	hs	n/a	Cell Signaling	7076	1:10,000 (5% milk in TBS-T)

## Data Availability

Not applicable.

## Supplementary Materials

The following supporting information can be downloaded at: https://www.mdpi.com/article/10.3390/ijms232113618/s1.
