# Supplementary figures and images for: Dynamin-like Protein 1 (DNML1) as a Molecular Target for Antibody-Based Immunotherapy to Treat Glaucoma
Source: Int J Mol Sci. 2022 Nov 7;23(21):13618. doi: 10.3390/ijms232113618 (PMC9654827; doi:10.3390/ijms232113618)

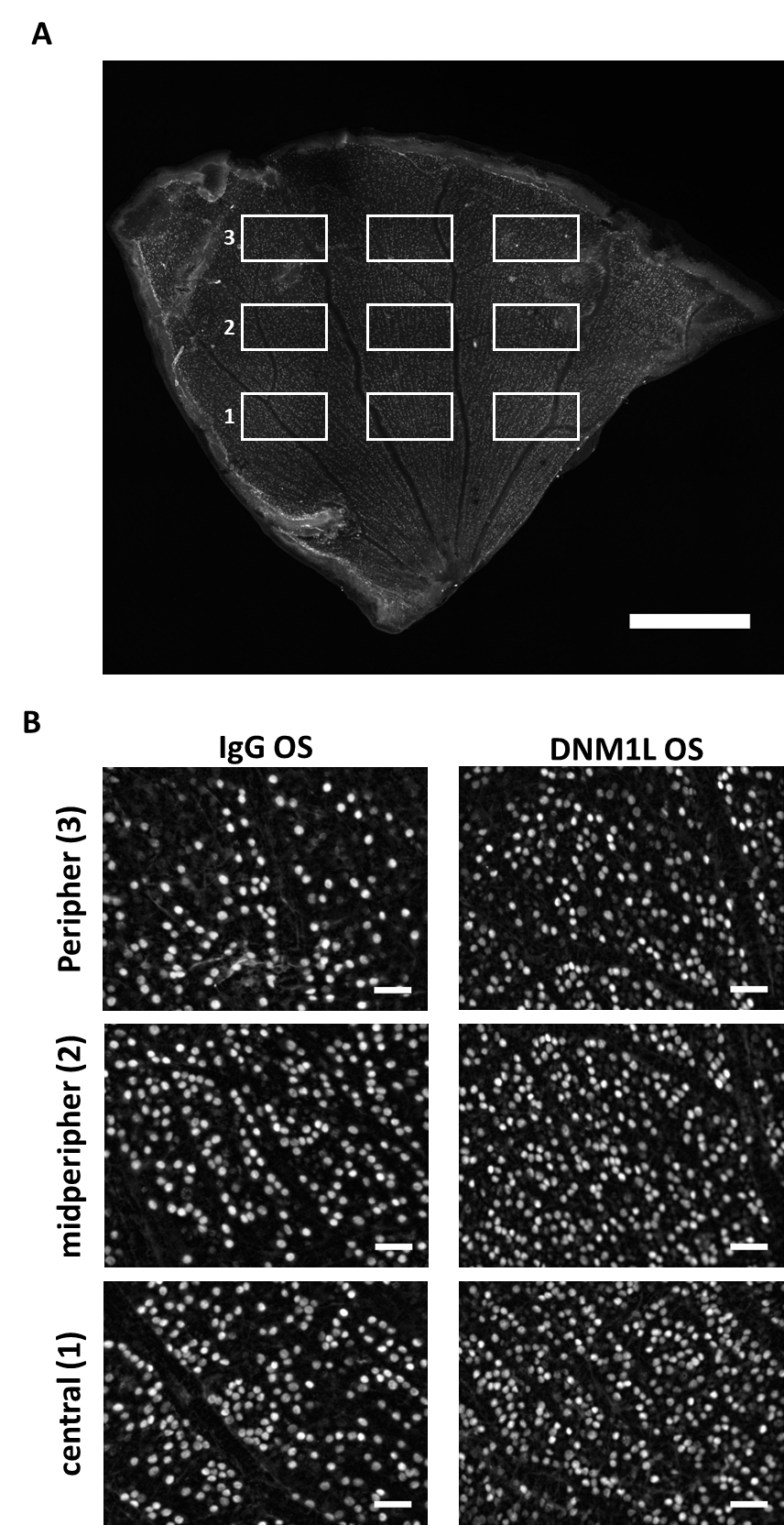

Supplement: Supplementary file 1 [file ijms-23-13618-s001.zip › Figure A1.tif]

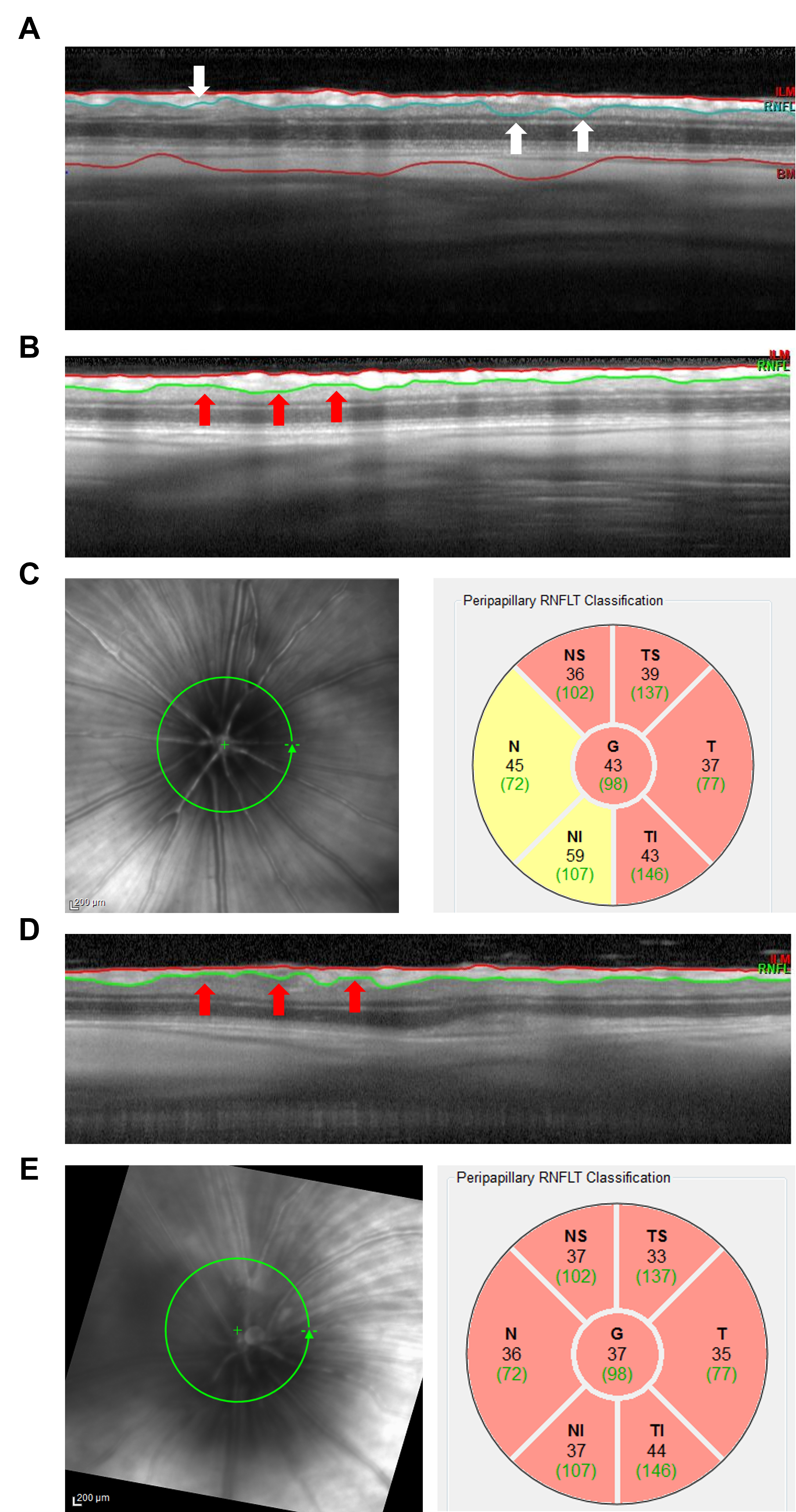

Supplement: Supplementary file 1 [file ijms-23-13618-s001.zip › Figure A2.tif]
